# An unusual presentation of chronic lymphocytic leukemia/small lymphocytic lymphoma on mammography: Case report

**DOI:** 10.1002/ccr3.4449

**Published:** 2021-07-06

**Authors:** Anas Mohamed, Ahmed I. Younes, Stephen Stalls, Aisha Kousar, Tian Li

**Affiliations:** ^1^ Department of Pathology and Laboratory Medicine East Carolina University/Vidant Medical Center Greenville NC USA; ^2^ Faculty of Medicine Omar Almukhtar University Albaida Libya

**Keywords:** breast, CLL/SLL, ductal carcinoma in situ, mammography, radiation

## Abstract

Although rare, breast CLL/SLL should be considered in the differential diagnosis of a breast mass. A high index of suspicion is needed to differentiate this neoplasm from more common breast carcinomas like solid variant of invasive lobular carcinoma.

## Graphical abstract

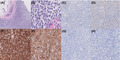

## 1 INTRODUCTION

Involvement of breast by chronic lymphocytic leukemia/small lymphocytic lymphoma (CLL/SLL) is exceedingly rare. While the final diagnosis of this entity is only possible after pathological examination and immunohistochemical stain utilization, a high index of suspicion is required to include it in the differential diagnosis of clinically or radiographically detectable breast lesions.

To date, five cases have been reported describing the involvement of the breast tissue by CLL/SLL. However, CLL/SLL in those patients was detected as a concurrent tumor with breast carcinoma^1^, ^2^ or as an abnormal palpable mass due to sentinel lymph node infiltration.^3^, ^4^ Our patient presented with a unilateral cluster of masses that was restricted to the upper outer quadrant of the right breast. The lesion was neither palpable nor painful, and there were no associated overlying skin changes or axillary lymphadenopathy.

## 2 CASE HISTORY

**FIGURE 1:**
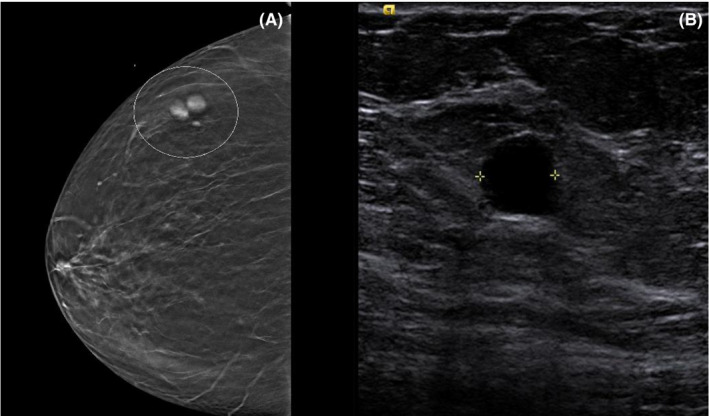
(A) A Cluster of radiopaque masses confined to superior lateral quadrant of right breast. (B) Ultrasonography image of the hypoechoic area that was biopsied and revealed the diagnosis of CLL/SLL

A 59‐year‐old African American woman with no significant past medical history was referred to our hospital for a suspicious lesion within the right breast discovered by a routine mammography scan. She was diagnosed with intermediate‐grade ductal carcinoma in situ (DCIS) of the left breast 13 years before presentation, which was positive for ER, PR, and negative for HER2. She was treated by partial mastectomy, followed by a combined treatment of whole‐breast radiation and antihormonal therapy (tamoxifen), with no reconstructive surgery performed. Her new lesion appeared as a focal area of asymmetry confined to the upper outer quadrant of the right breast (Figure 1A). Targeted ultrasonography was performed and revealed a cluster of multiple prominent lesions, the largest of which was 1 cm in diameter (Figure 1B); this finding was categorized as BIRADS 4. Considering her remote history of DCIS, the differential diagnosis of these findings included multi‐focal breast carcinoma and intraparenchymal lymph node metastasis. Therefore, an ultrasound‐guided biopsy was performed.

**FIGURE 2:**
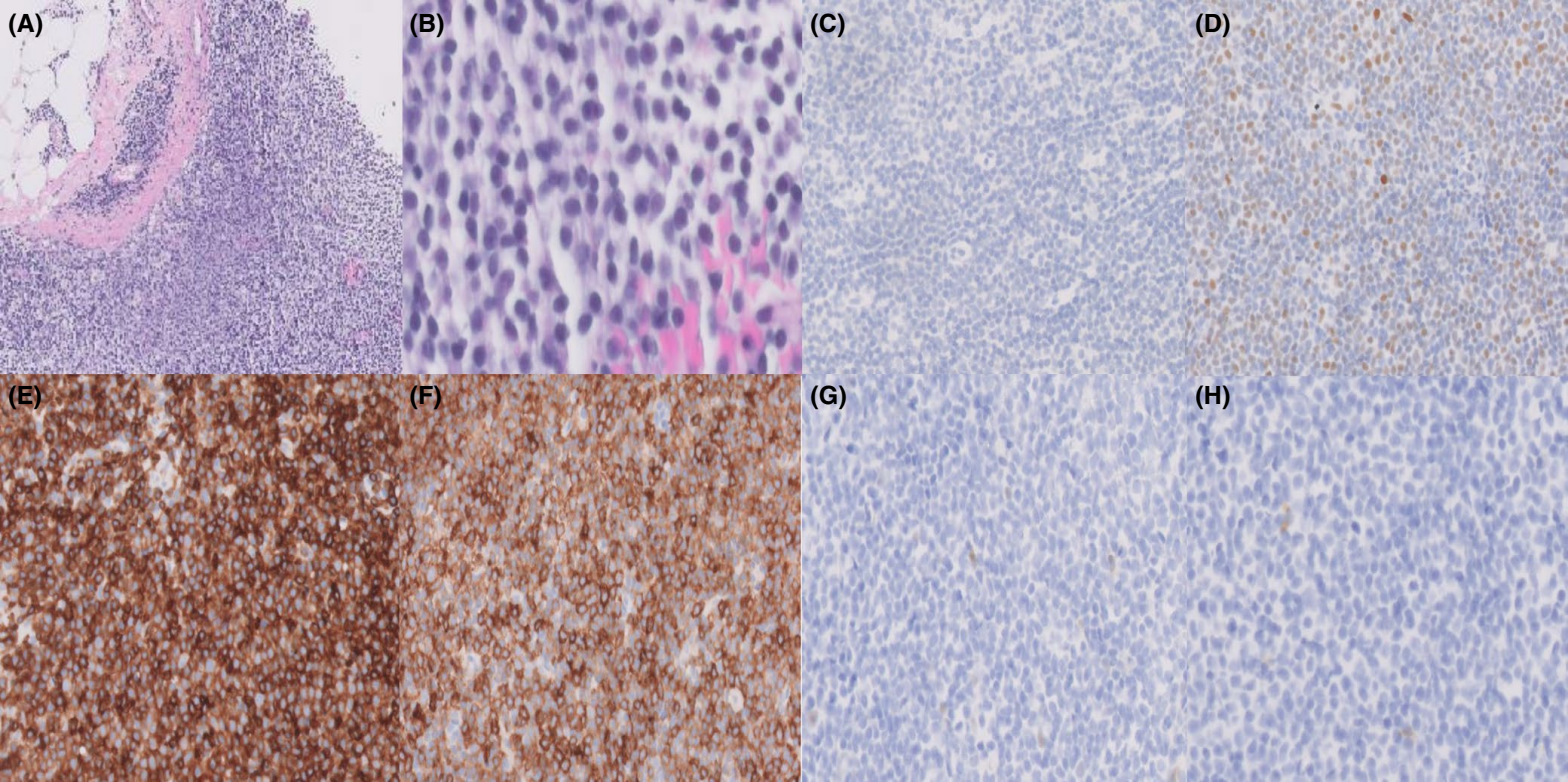
(A) Hematoxylin and eosin (H&E) staining shows breast tissue replacement by solid sheets of tumor cells with a background of adipose tissue (40×). (B) Small dark round lymphocytes on H&E (400×). (C) Tumor cells exhibit negative staining with pan‐cytokeratin (200×). (D) Lack of strong nuclear positivity for BCL6 (200×). (E) Neoplastic cells are positive for CD5 (200×). (F) Neoplastic cells are positive for CD23 (200×). (G) Tumor cells exhibit negative staining for BCL1 (200×). (H) CD10 is negative in neoplastic cells (200×)

The lesion demonstrated solid hypercellular sheets of small dark basophilic cells within a background of benign breast tissue (Figure 2A,B). No abnormal vascularity was observed. Primary and metastatic breast carcinoma were ruled out by the entirely negative staining pattern within the tumor cells using pan‐cytokeratin (Figure 2C). A low‐grade pattern of KI‐67 was seen with higher proliferation centers. While BCL6 was weakly positive in a subset of the neoplasm, strong nuclear positivity was not observed (Figure 2D). The aggregates of small neoplastic cells were positive for CD5 (Figure 2E), CD20, PAX5, BCL2, CD21, CD23 (Figure 2F), and CD43, and negative for BCL1 (Cyclin D1) (Figure 2G) and CD10 (Figure 2H). Moreover, fluorescent in situ hybridization (FISH) was negative for Cyclin D1. An initial complete blood count with a differential leukocytic count demonstrated WBC of 30 K/μl, with 69% lymphocytes, which increased to 59 K/μl during the follow‐up. Her platelet count was 225 K/μl, and her hemoglobin was 13.5 g/dl. Monocytosis and smudge cells were also detected. A computed tomography (CT) scan was performed and demonstrated moderate adenopathy within the lower neck, chest, abdomen, and pelvis. The overall clinical, radiographic, laboratory, and pathological findings were consistent with breast involvement by CLL/SLL. Based on Rai‐Sawitsky staging system, she is considered to be in the intermediate‐risk group. Hence, chemotherapy was not indicated. She remains asymptomatic. Apart from increased lymphocytosis, no evidence of disease progression is detected despite close follow‐up.

## 3 DISCUSSION

Leukemic or lymphoma involvement of the breast could be easily misinterpreted as an invasive carcinoma on imaging due to their very low incidence or their presentation together with coexisting breast cancer.^5^ Therefore, the importance of distinguishing between breast lymphoma and carcinoma conditions has recently emerged. Although mammography has been proven to be highly sensitive in detecting breast carcinoma, breast lymphoma cannot be solely diagnosed based on imaging.^6^ In our case, the lesion appeared as a round or oval mass, which has been observed in the majority of breast lymphoma cases.^7^ Other less common radiological findings include asymmetry, mammographically detectable lymphedema, and skin thickening.^7^ In contrast to breast carcinoma, breast lymphoma rarely, if ever, depicts microcalcifications on imaging.^7^ Therefore, different studies have stated the importance of various imaging modalities, such as MRI, to differentiate invasive breast carcinoma versus other hematologic malignancies of the breast.^1^

Microscopic evaluation revealed small, monomorphic, mature lymphocytes with no evidence of normal lymph node architecture, including the capsule. Variable pale proliferation centers were seen where mitotic activity was higher compared to other areas. Ki‐67 confirmed the mitotic activity spread. The lymphocytes were positive for CD5, CD20, PAX5, CD23, and CD43, which are the most consistent markers for CLL/SLL,^8^ while they were negative for germinal center markers such as CD10 and BCL6. Although the presence of breast carcinoma in our case was excluded by the negative expression of pan‐cytokeratin, a high index of suspicion is essential to distinguish between breast CLL/SLL and some histologic subtypes of carcinoma. For instance, solid variant of invasive lobular carcinoma can exhibit small, regular sized, discohesive cells arranged in sheets,^9^ which can morphologically mimic CLL/SLL. Furthermore, the absence of CCND1 on FISH excluded the majority of mantle cell lymphoma subtypes, which was confirmed with negative BCL1.^10^

Hematologic malignancies of the breast tend to present differently based on their classification. For instance, acute myelogenous leukemia could present as granulocytic myeloid sarcoma,^11^ while acute lymphocytic leukemia of the breast could present as a diffuse bilateral process with lymphadenopathy.^12^ Our patient's peripheral blood exhibited lymphocytosis with characteristic smudge cells (basket cells) which is a typical feature of CLL/SLL.

Our patient previously received whole‐breast irradiation with a total dose of 5040 cGy; previous reports showed that radiation might be absorbed in the contralateral breast from the scattered external beam.^13^ Physical DNA damage caused by radiation can activate tumor suppressor p53, which was proven to cause radiation‐induced lymphoma.^14^ Hence, we hypothesize that there might be a connection between her CLL/SLL diagnosis and her past medical history, which would need further research to confirm.

Finally, despite being rare, it is essential to consider CLL/SLL in the differential diagnosis of unilateral irregular breast aggregates on mammography. It is an uncommon entity that should be further studied, particularly in patients with a history of DCIS or those who receive radiation treatment, even if it is localized. Her CLL/SLL was discovered on imaging before she developed any systemic manifestations, which is also uncommon.

## 4 CONCLUSION

In conclusion, our patient developed CLL/SLL, which was confined to the right breast and was not associated with any breast carcinoma. Awareness of this patient's profile, particularly the long duration between DCIS treatment and the development of CLL/SLL, could give insight into the value of long‐term surveillance for such patients after recovery. It also highlights the role of imaging techniques and pathological evaluation in recognition of breast leukemia and lymphoma.

## CONFLICTS OF INTEREST

All authors declare no conflict of interest.

## AUTHOR CONTRIBUTIONS

Anas Mohamed and Ahmed I Younes: Drafted and wrote the article and designed figures. Stephen Stalls: Edited and reviewed the article. Aisha Kousar: Edited and reviewed the article. Tian Li: Revised the manuscript critically and provided final suggestions for final preparation.

## ETHICS STATEMENT

This manuscript is in compliance with the ethical standards of the Institutional Review Board policy of East Carolina University and with the 1964 Helsinki Declaration and its later amendments.

## Data Availability

The data that support the findings of this study are available on request from the corresponding author. The data are not publicly available due to privacy or ethical restrictions.
